# Predicting cancer involvement of genes from heterogeneous data

**DOI:** 10.1186/1471-2105-9-172

**Published:** 2008-03-27

**Authors:** Ramon Aragues, Chris Sander, Baldo Oliva

**Affiliations:** 1 Structural Bioinformatics Lab. (GRIB), Universitat Pompeu Fabra-IMIM, Barcelona Research Park of Biomedicine (PRBB), 08003-Barcelona, Catalonia, Spain; 2 Computational Biology Center, Memorial Sloan-Kettering Cancer Center, 1275 York Avenue, Box 460, New York, NY 10065, USA

## Abstract

**Background:**

Systematic approaches for identifying proteins involved in different types of cancer are needed. Experimental techniques such as microarrays are being used to characterize cancer, but validating their results can be a laborious task. Computational approaches are used to prioritize between genes putatively involved in cancer, usually based on further analyzing experimental data.

**Results:**

We implemented a systematic method using the PIANA software that predicts cancer involvement of genes by integrating heterogeneous datasets. Specifically, we produced lists of genes likely to be involved in cancer by relying on: (i) protein-protein interactions; (ii) differential expression data; and (iii) structural and functional properties of cancer genes. The integrative approach that combines multiple sources of data obtained positive predictive values ranging from 23% (on a list of 811 genes) to 73% (on a list of 22 genes), outperforming the use of any of the data sources alone. We analyze a list of 20 cancer gene predictions, finding that most of them have been recently linked to cancer in literature.

**Conclusion:**

Our approach to identifying and prioritizing candidate cancer genes can be used to produce lists of genes likely to be involved in cancer. Our results suggest that differential expression studies yielding high numbers of candidate cancer genes can be filtered using protein interaction networks.

## Background

Tumor development results from a progressive sequence of genetic and epigenetic alterations that promote the malignant transformation of the cell by disrupting key processes involved in normal growth control and tissue homeostasis [1]. Since complex biological networks control these processes, there are many genes that, mutated, can provide the cell with a specific aberrant capability. Alterations in three types of genes are responsible for tumorigenesis: oncogenes, tumor-suppressor genes, and stability genes [2]. Most oncogenes are involved in controlling the rate of cell growth, while tumor suppressor genes are usually negative regulators of growth or other functions that may affect invasive and metastatic potential, such as cell adhesion and regulation of protease activity. On the other hand, stability genes control the rate of DNA mutation, and their alteration can result in mutations in oncogenes or tumor suppressor genes, thus contributing to the development of cancer [3].

The completion of the human genome project and the development of high-throughput experimental techniques have enabled new approaches for studying cancer. For example, gene-expression profiling using microarrays has improved the classification of some tumor types [4,5]. Moreover, data from large-scale screenings of protein-protein interactions has been used to identify interaction subnetworks activated in cancer [6]. Finally, genome scanning for gene copy-number alterations has detected many loci harboring candidate cancer genes [7]. Because of these advances, efforts to catalog all of the mutational events that contribute to human cancer can now be envisioned. For example, the Cancer Genome Atlas initiative [8] is resequencing a substantial fraction of human genes in order to elucidate the contribution of somatic mutations to cancer development and progression. Due to the complexity of these initiatives, methods to characterize and prioritize gene candidates likely to be involved in cancer are being developed [9-12].

Protein interaction networks are a useful tool for better understanding the biology of the cell [13-15]. Moreover, the topology of the networks and the neighborhood of a given protein within the network have been used to functionally characterize proteins [16,17]. It has also been observed that proteins related to a disease tend to have a high connectivity between them [18], specifically in inherited diseases [19,20] and ataxia [21]. Moreover, in a recent work by Barabasi and coworkers, somatic cancer genes (i.e., those that are not transmitted to descendants) were found to be more likely than other genes to encode proteins with many interaction partners (i.e., hubs) [18].

Gene expression profiling with DNA microarrays is a powerful approach for identifying cancer genes. Numerous studies have presented analyses of human cancer samples in which they identify gene expression signatures for different cancer types and subtypes [22-24]. In these experiments, genes are ranked according to their differential expression in the majority of cancer samples with respect to normal tissues, and genes above a predefined threshold are considered as candidate genes for the type of cancer being studied. Often, more in-depth analyses are performed to evaluate the involvement of candidate genes in the cancer, either by means of proteomics techniques [25], real-time polymerase chain reaction (qRT-PCR) [26], or literature search [27]. However, validating the results of microarray experiments can be a long and costly effort, due to the large number of candidate genes typically involved. Often, only a handful of genes of interest are selected for experimental validation, and hundreds of others are ignored. Moreover, due to limitations in DNA microarray technology, higher differential expressions of a gene do not necessarily reflect a greater likelihood of the gene being related to cancer [28] and therefore, focusing only on the candidate genes with the highest differential expressions might not be the optimal procedure. Thus, there is a need for better techniques for selecting which genes will be analyzed in detail. Several procedures address the issue of selecting genes related to cancer [29] by further processing microarray data, either using more powerful statistics [30] or integrating multiple expression studies [31].

In order to improve the candidate gene selection process, several works have combined gene expression with other types of genomic data [32,33]. One popular approach is gene set enrichment analysis, in which statistical tests are used to identify sets of dysregulated genes with a common biological function [34,35]. Recently, Chinnaiyan and coworkers have combined the Molecular Concept Map and expression signatures to profile prostate cancer progression from benign epithelium to metastatic disease [36]. In the work of Rhodes *et al*. [6], instead of relying on predefined gene annotations, they applied a human interactome to genome-wide gene expression data in cancer for identifying a potential tumor suppressor gene in the integrin signaling pathway, and then demonstrated the utility of protein-protein interaction data for identifying interaction subnetworks activated in cancer. Finally, other approaches avoid the use of high throughput data by predicting cancer genes candidates based on their sequence, structure and functional properties [9,37].

Here, we have implemented a systematic approach for identifying genes (and gene products) involved in cancer. Our method produces lists of reliable candidate cancer genes by combining (i) a list of known cancer genes [11]; (ii) protein-protein interaction data [38]; (iii) expression information from multiple cancer studies [39]; and (iv) probabilities derived from structural, functional and evolutionary properties [37]. We begin by evaluating each method separately and comparing their results. Next, we present the integrative approach and evaluate its potential for predicting cancer genes. We provide candidate cancer genes obtained as a result of this work and assess them using public repositories of biological information and literature search. We conclude by discussing potential applications of our method.

## Results

We were interested in assessing different methodologies for identifying cancer genes. Specifically, we tested the use of (i) protein interaction networks; (ii) microarray differential expression data; (iii) structural, functional and evolutionary properties of genes; and (iv) an integration of the three previous type of data. For the evaluation, we relied on a cancer gene list compiled from a variety of curated lists, cancer and sarcoma reviews, and Entrez Gene queries, followed by additional curation [11] (Material and Methods). We refer to genes annotated as "tumor suppressors", "oncogenes" or "stability genes" in this list as the known cancer genes. Moreover, we use the term "cancer genes" to refer to genes and proteins involved in cancer.

### Predicting cancer genes based on protein interaction partners

**Figure 1:**
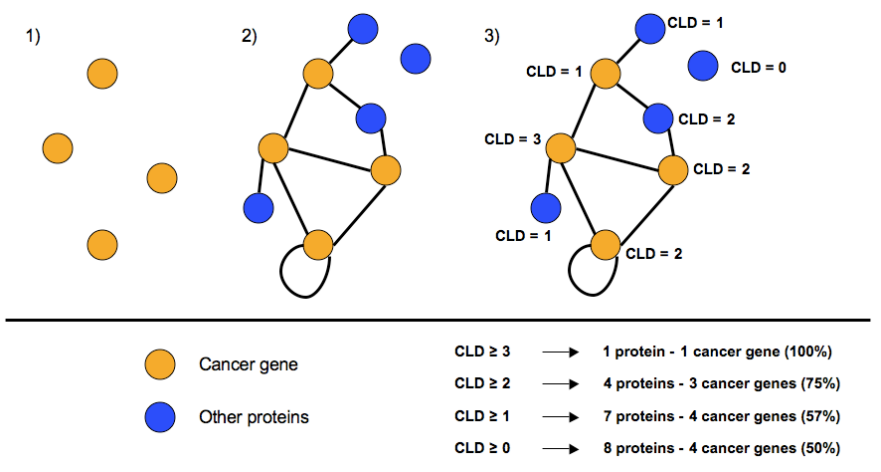
**Calculating the Cancer Linker Degree (CLD) of a protein**. The Cancer Linker Degree (CLD) of a protein is defined as the absolute number of partners of the protein that are known to be involved in cancer. The procedure followed to calculate the CLD of a protein consists of 3 steps: 1) setting the known cancer genes as seeds; 2) retrieving the direct interaction partners for the known cancer genes; and 3) calculating the CLD of each protein (i.e. the number of known cancer genes to which it is connected). In the example provided, we observe that proteins with high CLD are more likely to be cancer gene products that proteins with low CLD.

We assessed the use of protein interaction networks for predicting cancer genes. We hypothesized that proteins whose partners have been annotated as cancer genes are likely to be cancer genes as well: if a mutated gene is perturbing a pathway related to cancer (e.g. growth control), mutations to interaction partners are also likely to perturb the same pathway. As corollary, proteins with many interactions with cancer genes should be more likely to be involved in cancer than proteins with just one cancer gene partner. We used the PIANA (Protein Interactions And Network Analysis) tool [38] to build a cancer protein interaction network, using as seeds the gene products of the known cancer genes (Material and Methods). Thus, the cancer protein interaction network is composed of the known genes and their direct interaction partners. In this network, we define the cancer linker degree (CLD) of a protein as the number of cancer genes to which it is connected, excluding the protein itself (Figure 1). We examined the relationship between the CLD of a protein and its likelihood of being a known cancer gene, finding that that the cancer linker degree of a protein is a good indicator of the probability of being a cancer gene (Table 1). The significance of this observation (Table 1) was confirmed by both a Fisher's exact test and a permutation analysis (Methods). The latter was performed by using a Wilcoxon signed rank test to compare the ratio of cancer genes among proteins with CLD ≥ *threshold *to the percentage of cancer genes in 1000 random samples of *N *proteins with at least one interaction in PIANA (N being the number of proteins with CLD ≥ *threshold*).

**Table 1:** Cancer gene enrichment of proteins according to their Cancer Linker Degree. The enrichment of cancer genes is shown for proteins with CLD ≥ 0, CLD ≥ 1 and CLD ≥ 10. The p-value of the difference between the whole data set (proteins with CLD ≥ 0) and proteins with CLD ≥ 1 and CLD ≥ 10 was calculated using the Fisher's exact test for count data (F) and the Wilcoxon signed rank test (W) on 1000 random samples.

	proteins CLD ≥ 0	proteins CLD ≥ 1	*p*-value CLD ≥ 0 vs. CLD ≥ 1	proteins CLD ≥ 10	*p*-value CLD ≥ 0 vs. CLD ≥ 10	*p*-value CLD ≥ 1 vs. CLD ≥ 10
% of cancer genes	10%	15%	< 2.2 × 10^-16 ^**(F)** < 2.2 × 10^-16 ^**(W)**	48%	< 2.2 × 10^-16 ^**(F)** < 2.2 × 10^-16 ^**(W)**	< 2.2 × 10^-16 ^**(F)**

**Figure 2:**
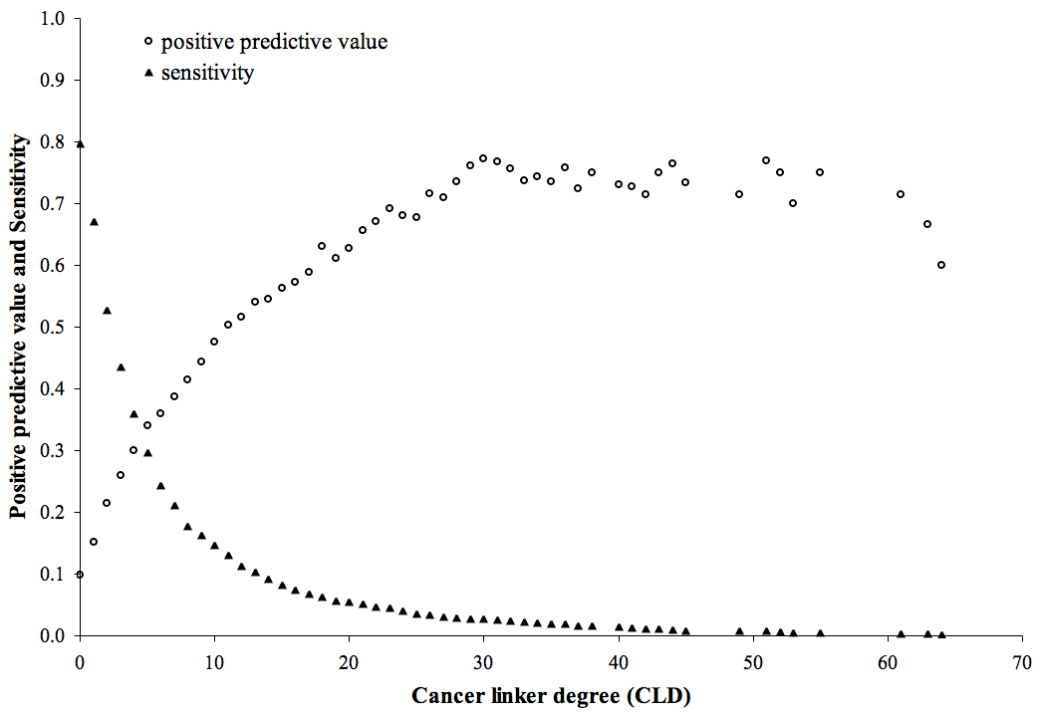
**Positive predictive value and Sensitivity when predicting cancer genes based on the cancer linker degree of proteins**. The positive predictive value and sensitivity shown are for accumulative cancer linker degrees (CLD) (i.e. cancer linker degree 5 represents proteins with CLD ≥ 5). The average protein in the data set is represented by CLD 0.

Furthermore, we used the cancer linker degree of proteins to predict cancer genes (Methods), obtaining a positive predictive value of ~54% at sensitivity of ~10% (Figure 2). We studied the robustness of this method to variations in the input cancer gene list by i) randomly removing 10%, 25%, 50% and 75% of proteins from the set of known cancer genes; and ii) using a different input cancer gene list [40]. In the first case (Additional file 1), the removal of 10% or 25% of the proteins did not affect the high positive predictive value obtained when using the complete input list. Removing 50% or 75% of input cancer genes decreased the positive predictive value, but this remained higher for proteins with CLD ≥ 1 than that of the average protein from the dataset. In the second case (Additional file 2), using a different input list of known cancer genes obtained a positive predictive value of 10% for proteins with CLD ≥ 1, which is significantly higher than the 6% obtained for proteins with CLD ≥ 0 (*p*-value < 2.2 × 10^-16^).

The CLD of a protein depends on the number of interactions that have been reported for the protein and thus, it might be influenced by how much interest has been placed on a protein by the research community. To exclude this potential bias we calculated the cancer linker degree of proteins i) using only interactions from high-throughput studies (i.e yeast two hybrid and affinity purification systems); and ii) using all interactions in PIANA except for those in the Human Protein Reference Database [41], which is a manually curated database of interactions extracted from the literature, with a preference towards disease related proteins. In the first case, we observed a decrease in positive predictive value (Additional file 3), while in the second scenario there was a slight increase in the positive predictive value (Additional file 4). In both cases, there is a significant enrichment of proteins with CLD ≥ 1 with respect to the average protein in the dataset (*p*-value of 4.8 × 10^-14 ^and *p*-value < 2.2 × 10^-16^, respectively), concluding that the literature bias does not invalidate our initial hypothesis. Besides, similarly to previous studies [18,42], we observed that proteins with a large number of interaction partners (i.e., hubs) are more likely to be cancer genes than proteins with few interaction partners (Additional file 5). However, using the total number of interacting partners of a protein to predict cancer genes performed worse than using the cancer linker degree: for sensitivity of ~10%, the positive predictive value was ~34%.

### Predicting cancer genes based on microarray data

**Figure 3:**
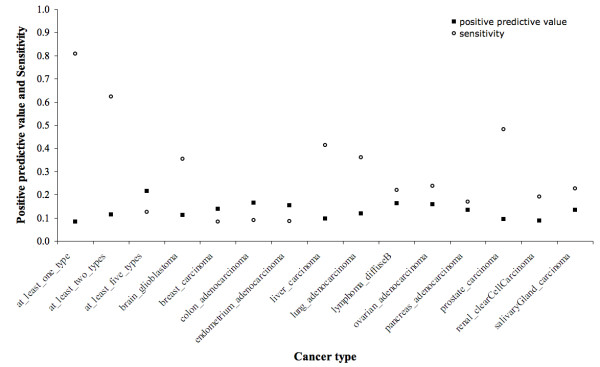
**Positive predictive value and sensitivity when predicting cancer genes based on differential expression data**. The positive predictive value and sensitivity are shown for 12 cancer types and genes over- or under-expressed in at least 1, 2 and 5 cancer types.

We evaluated the use of differential expression data to predict cancer genes. We based our study on Oncomine [39] lists of over- and under-expressed genes in 24 differential expression studies, which we manually grouped in 12 different cancer types (see Material and Methods and Additional file 6). The positive predictive value was between 9–16% for all cancer types, with sensitivity ranging from 84% (for genes over- or under-expressed in at least one cancer type) to 8% (for breast cancer) (Figure 3). In contrast, only 4% of human genes from our dataset were found to be known cancer genes. We confirmed the significance of this observation by performing the Fisher's exact test for count data and the Wilcoxon signed rank test on the enrichment of cancer genes on 1000 random samples of N human genes (N being the number of genes appearing differentially expressed in at least X cancer types). We also observed that genes appearing differentially expressed in multiple cancer types are significantly more likely to be known cancer genes than those appearing differentially expressed in just one cancer type (Table 2). For example, 22% of genes found differentially expressed in at least 5 cancer types are cancer genes, compared to 8% of genes found differentially expressed in at least one cancer type. These results confirm the need for post-processing in differential expression studies: microarrays detect many cancer genes, but they are usually mixed with many non-cancer genes.

**Table 2:** Cancer gene enrichment of proteins according to the number of cancer types in which they appear differentially expressed. The enrichment of cancer genes is shown for proteins differentially expressed in 1, 2 and 5 cancer types. The *p*-value of the difference between the different groups of proteins was calculated using the Fisher's exact test for count data (F) and the Wilcoxon signed rank test (W) on 1000 random samples.

	All in dataset	1 cancer type	2 cancer types	5 cancer types
% of cancer genes	4%	8%	11%	22%
		*p*-values	*p*-values	*p*-values
		all vs. 1 < 2.2 × 10^-16 ^(W)	all vs. 2 < 2.2 × 10^-16 ^(W)	all vs. 5 < 2.2 × 10^-16 ^(W)
			1 vs. 2 = 2.6 × 10^-11 ^(F)	1 vs. 2 = 2.6 × 10^-11 ^(F)
				2 vs. 5 = 2.0 × 10^-13 ^(F)

Moreover, we studied the effect of looking at over- and under-expressed genes by their differential expression rank in a given experiment (Methods). For each differential expression study, we calculated the enrichment of cancer genes among i) the 100 most differentially expressed genes; and ii) all differentially expressed genes. None of the 24 experiments tested showed a significant increase in positive predictive value when restricting the predictions to the 100 most differentially expressed genes. These results suggest that the number of cancer types in which a gene is observed differentially expressed is a better strategy for predicting cancer genes than using its differential expression rank.

### Predicting cancer genes by structural, functional and evolutionary properties

**Figure 4:**
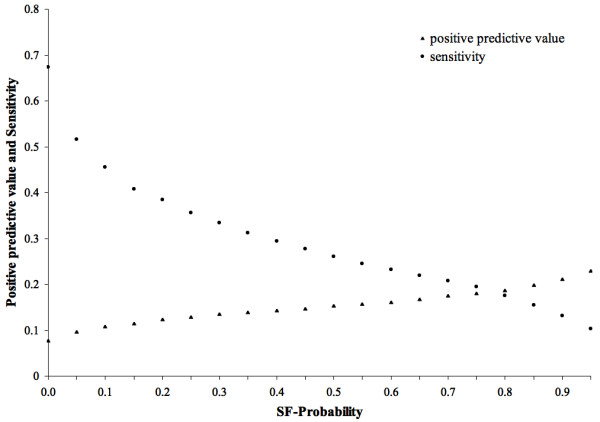
**Positive predictive value and sensitivity when predicting cancer genes based on their probability of being a cancer gene according to structural, functional and evolutionary properties (SF-Probability)**. The positive predictive value and sensitivity shown are for accumulative SF-Probabilities (i.e. SF-Probability 0.7 represents genes with SF-Probability ≥ 0.7). The average gene in the data set is represented by SF-Probability ≥ 0. SF-Probabilities were obtained from [37].

Cancer genes have been shown to have common structural, functional and evolutionary properties [9,37] and therefore, the properties of a gene can be used to estimate its probability of being a cancer gene [37]. We used the results from the work of López-Bigas and coworkers [37] to calculate the positive predictive value and sensitivity when predicting cancer genes based on the structural, functional and evolutionary properties of genes (hereafter, we refer as SF-Probabilities to the probabilities assigned to genes in [37]). As shown on Figure 4, SF-Probabilities higher or equal to 0.90 yielded a positive predictive value of 21% at sensitivity of 13%, while for the average protein in the dataset (i.e. proteins with SF-Probability ≥ 0) the positive predictive value was 8% at sensitivity of 67%. Moreover, the observed greater enrichment of cancer genes among proteins with SF-Probability ≥ 0.1 with respect to the average protein in the data set is significant (11% versus 8%, *p*-value of 1.1 × 10^-10^).

### Relating the Cancer Linker Degree to differential expression and SF-Probability

#### Proteins with a high cancer linker degree tend to be differentially expressed in multiple cancer types

**Figure 5:**
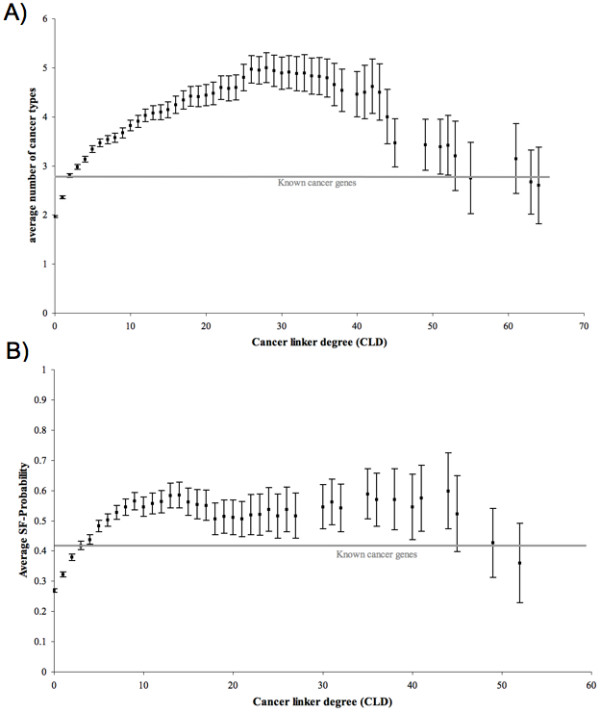
**The average number of cancer types in which genes appear differentially expressed (A) and the probability of being a cancer gene according to structural, functional and evolutionary properties (B) are plotted as a function the cancer linker degree (CLD) of the gene products**. A) The average number of cancer types shown are for an accumulative CLD (i.e. CLD 5 represents proteins with CLD ≥ 5). The average protein in the dataset is represented by CLD 0. Known cancer genes appear differentially expressed in an average of 2.8 cancer types. B) The average SF-Probabilities shown are for an accumulative CLD (i.e. CLD 5 represents proteins with CLD ≥ 5). The average protein in the dataset is represented by CLD 0. Known cancer genes had an average SF-Probability of 0.41.

We were interested in examining the relationship between the cancer linker degree (CLD) of a protein and the number of cancer types in which its corresponding gene was differentially expressed. If proteins with high CLD tended to be differentially expressed in more cancer types than other proteins, that would suggest an involvement of high-CLD proteins in cancer. We observed that proteins with high CLD are significantly more likely to be found differentially expressed in multiple cancer types than the average protein in the dataset (Figure 5A). For example, proteins with CLD ≥ 1 appear differentially expressed in an average of 2.4 cancer types, which is significantly higher than for proteins with CLD ≥ 0 (1.96 cancer types, p-value < 2.2 × 10^-16^), but significantly lower than for proteins with CLD ≥ 20 (4.4 cancer types, *p*-value < 2.2 × 10^-16^). Furthermore, known cancer genes are found over- or under-expressed in an average of 2.8 cancer types.

#### Proteins with a high cancer linker degree tend to have common functional, structural and evolutionary properties with cancer genes

We tested the correlation between the cancer linker degree (CLD) of proteins and their probabilities of being cancer genes according to their structural, functional and evolutionary properties (SF-Probabilities). We observed a significant difference between the SF-Probabilities of random proteins from the database (i.e. proteins with CLD ≥ 0) and the SF-Probabilities of proteins with interactions to cancer genes (Figure 5B). For example, we found that proteins with CLD ≥ 1 had an average SF-Probability of 0.32, which is significantly higher than for proteins with CLD ≥ 0 (SF-Probability of 0.27, p-value = 1.3 × 10^-9^) but significantly lower than for proteins with CLD ≥ 20 (SF-Probability of 0.51, p-value = 0.001). The lower SF-Probability of proteins with very high CLDs is explained by the few cases found with multiple interactions to known cancer genes. These results suggest that proteins with interactions to cancer genes show structural, functional and evolutionary properties similar to cancer genes.

### Predicting cancer genes by integrating multiple types of data

**Figure 6:**
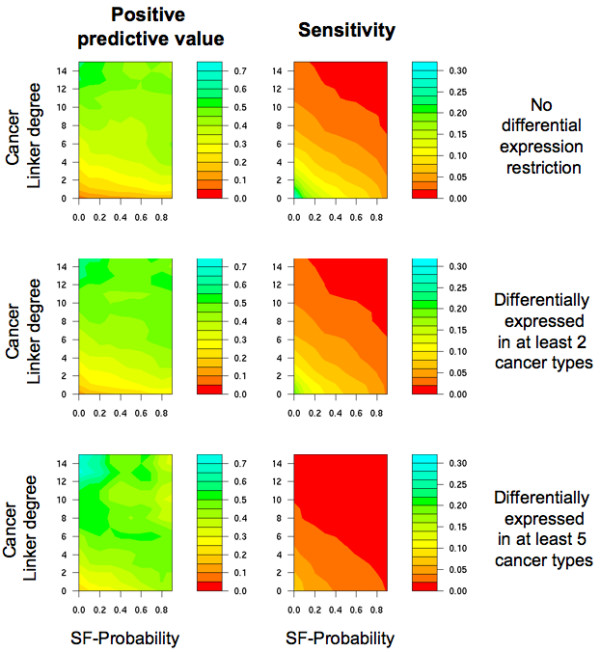
**Contour maps for positive predictive value and sensitivity obtained when varying the thresholds applied by the integrative approach**. In each of the following images, the x-axis is the SF-Probability threshold and the y-axis is the cancer linker degree (CLD) threshold. For a given restriction on the number of cancer types in which a gene must be differentially expressed in order to be considered a candidate (no restriction, at least two cancer types and at least 5 cancer types), the positive predictive value and sensitivity are provided for each combination of CLD and SF-Probability. Positive predictive values and sensitivities are shown using colored contour maps, from red (i.e. 0) to turquoise (i.e., 0.7 for positive predictive value and 0.3 for sensitivity). For example, imposing a gene to be differentially expressed in at least two cancer types, with a CLD of 6 and with an SF-Probability of 0.4, the positive predictive value is 0.4 for sensitivity of 0.05.

**Figure 7:**
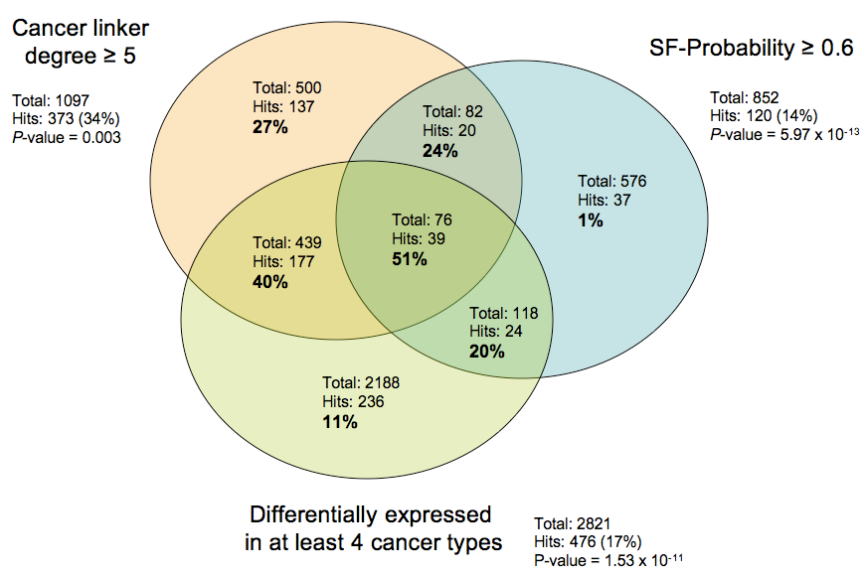
**Positive predictive value calculated for diverse overlaps of cancer gene candidates**. The criteria applied was the following: (i) cancer linker degree ≥ 5; (ii) differentially expressed in at least four cancer types; and (iii) SF-Probability ≥ 0.6. The Venn diagram shows the total number of candidates, the number of hits (i.e. known cancer genes among the candidates) and the positive predictive value for overlap case. For example, the positive predictive value when solely applying an SF-Probability threshold of 0.6 was 14%. In contrast, when combining the SF-Probability with a cancer linker degree threshold of 5, the positive predictive value was 37% (59 hits for a total of 158 candidates).

We evaluated the approach that predicts cancer genes by taking into account three different methodologies: 1) the cancer linker degree (CLD) of proteins; 2) the number of cancer types in which a gene appears differentially expressed with respect to normal tissue; and 3) the probability of being a cancer gene according to structural, functional and evolutionary properties (SF-Probability) [37]. First, each methodology was applied independently, obtaining three different scores for each human gene. Next, for each possible combination of score thresholds, a list of cancer gene candidates was produced by selecting genes that respected the three thresholds. The positive predictive values of this integrative approach range from 23% at sensitivity of 15% (for CLD ≥ 1, differentially expressed in at least one cancer type and SF-Probability ≥ 0.1) to 73% at sensitivity of 1% (for CLD ≥ 15, at least 5 cancer types and SF-Probability ≥ 0.0). Figure 6 shows the positive predictive value and sensitivity obtained when using multiple combinations of thresholds. The two criteria that most contribute towards obtaining high positive predictive values are the CLD threshold and the number of cancer types in which a gene must be differentially expressed. We also studied the difference between using the integrative approach and applying the CLD method alone (Table 3), observing that the integrative approach should be used when high CLD thresholds cannot be applied (e.g., not enough interaction information is available). For example (Figure 7), the positive predictive value for each type of data used independently is (i) 34% for proteins with CLD ≥ 5; (ii) 17% for genes differentially expressed in at least 4 cancer types; and (iii) 14% for SF-Probability ≥ 0.6, while the combined use of these three thresholds obtains a significantly greater positive predictive value of 51% (*p*-values of 0.003, 1.53 × 10^-11 ^and 5.97 × 10^-13^, respectively).

**Table 3:** Comparing the performances of the integrative approach and the Cancer Linker Degree method. Positive predictive values (PPV) and sensitivities are shown under nine different fixed cancer linker degrees (CLD) for a method solely based on CLD scores and an integrative approach which combines the CLD score with SF-Probability and the number of cancer types in which the gene appears differentially expressed. For all CLD thresholds above 3, the difference between the integrative approach and the CLD method alone is not significant. The *p*-value of the difference between the two different groups of cancer gene candidates was calculated using the Fisher's exact test.

	**CLD alone**		**Integrative approach** **• SF-Probability ≥ 0.3** **• No. Cancer types ≥ 1**		***P*-value**
		
	**PPV**	**Sensitivity**	**PPV**	**Sensitivity**	
CLD ≥ 1	15%	67%	26%	11%	4.2 × 10^-9^
CLD ≥ 2	21%	53%	28%	9%	0.005
CLD ≥ 3	26%	44%	32%	8%	0.035
CLD ≥ 4	30%	36%	34%	6%	0.194
CLD ≥ 5	34%	30%	39%	6%	0.245
CLD ≥ 10	48%	15%	43%	3%	0.451
CLD ≥ 15	56%	8%	46%	1%	0.272
CLD ≥ 20	63%	5%	58%	1%	0.799
CLD ≥ 25	68%	4%	75%	1%	0.744

### Cancer gene candidates

**Figure 8:**
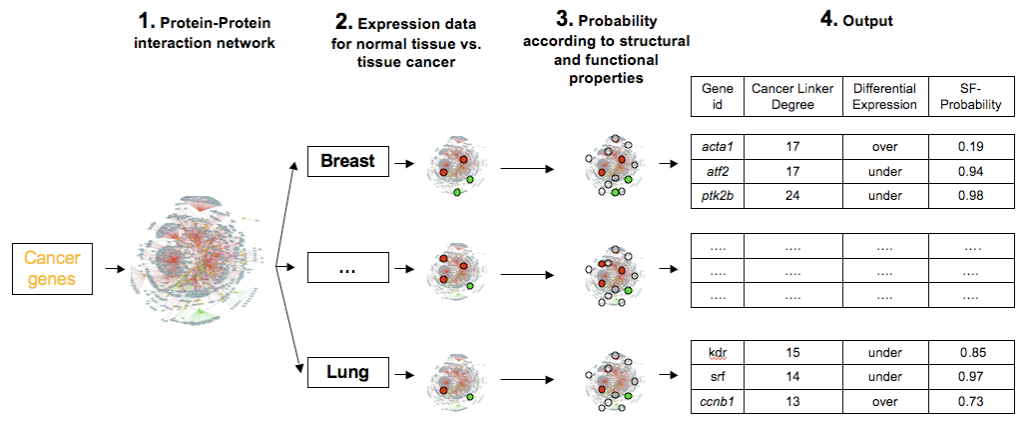
**Procedure followed to predict cancer gene candidates**. First, a cancer protein interaction network is built from the list of known cancer genes. Second, expression data from different cancer types is mapped onto the network. Third, probabilities of being a cancer gene based on structural, functional and evolutionary properties are retrieved for proteins in the network. Fourth, cancer genes are predicted based on the thresholds provided by the user for each type of data.

The procedure followed to predict cancer gene candidates consists of four steps (Figure 8 and Methods): (i) using PIANA [38] to build the protein interaction network by using the known cancer genes as seeds; (ii) mapping differentially expressed genes onto the network for each cancer type; (iii) mapping SF-Probabilities from [37] onto the network; (iv) producing an ordered list of candidates.

We provide the complete list of human cancer gene candidates for which at least one type of data indicated a relationship to cancer (Additional file 7). This list comprises 11,576 candidates, 1,040 of which scored in the three approaches (i.e., CLD > 0, differentially expressed in at least one type of cancer and SF-Probability > 0). We have also produced a short list of 20 candidate cancer genes (Table 4). Proteins in Table 4 have a cancer linker degree (CLD) equal or greater than 10, are differentially expressed in at least three cancer types and their SF-Probability is equal or greater than 0.7. We analyzed (Table 5) cancer gene candidates from Table 4 based on literature search [43] and descriptions from UniProt [44], Reactome [45] and the Gene Ontology (GO) [46]. This analysis suggests that our approach to identifying cancer genes is reliable: 60% of the proposed candidates have been directly related to cancer in experimental studies described in the literature, and an extra 25% participates in pathways known to be implicated in cancer. For example, the spleen tyrosine kinase (*syk*), predicted by the method to be a cancer gene, has been recently added (in a date subsequent to the creation of our list of known cancer genes) to the Sanger Cancer Gene Census [9]. *Syk*, with a cancer linker degree of 17, found differentially expressed in 4 types of cancer and with a SF-Probability of 0.99, is a positive effector of BCR-stimulated responses [47] and has been found to be involved in urinary bladder carcinoma [48] and primary liver cancer [49]. Besides, other candidate cancer genes have been very recently related to cancer in the literature (e.g., *mst1r*, involved in breast cancer [50]) or are known to be involved in pathways implicated in cancer (e.g. *srf *is a nuclear repressor of Smad3-mediated TGF-beta signaling [51], which induces apoptosis in numerous cell types). Finally, genes such as *surb7 *and *kin27 *were not found to be involved in cancer according to the literature and thus we suggest future experimental studies to focus on evaluating their potential involvement in cancer. Literature references for each cancer gene candidate found to be involved in cancer are provided as Additional file 8.

**Table 4:** Cancer gene candidates. The cancer gene candidates of this table where obtained by fixing the following thresholds: (i) cancer linker degree equal of higher than 10; (ii) found differentially expressed in at least three cancer types; and (iii) probability based on structural, functional and evolutionary properties (SF-Probability) equal of higher than 0.7.

**Gene name**	**Cancer Linker degree**	**Number of cancer types differentially expressed**	**SF-Probability**
CDK9	11	6	0.97
GATA2	10	5	0.99
ATF2	17	6	0.94
CCNB1	13	3	0.73
CSNK2A2	22	4	0.89
PPARBP	14	5	0.99
CSK	19	5	0.90
KIN27	35	6	0.82
CUL1	12	3	0.85
DKFZP686I18166	11	6	0.99
STAT5B	20	6	0.99
MCM7	14	4	0.99
SURB7	14	4	0.74
MST1R	10	4	0.74
KHDRBS1	17	6	0.92
SYK	17	4	0.99
KDR	15	4	0.85
NME2	11	5	0.99
POLR2B	12	3	0.82
SRF	14	7	0.97

**Table 5:** Analysis of predicted cancer genes in Table 4. Column "related to cancer" indicates whether literature [43] and information coming from UniProt [44], Reactome [45] and GO [46] indicate a strong involvement in cancer (++), somehow related to cancer (+) or not related to cancer (-). Literature references for each gene found to be involved in cancer are provided as additional file 8.

**Gene name**	**Description and Function/Pathway**	**Related to cancer**
CDK9	Cell division protein kinase 9 Regulation of progression through cell cycle	++
GATA2	Endothelial transcription factor GATA-2 Transcriptional activator which regulates endothelin-1 gene expression	+
ATF2	Cyclic AMP-dependent transcription factor ATF-2 Transcriptional activator which binds to the CRE, present in many viral and cellular promoters.	+
CCNB1	G2/mitotic-specific cyclin-B1 Essential for the control of the cell cycle at the G2/M (mitosis) transition.	++
CSNK2A2	Casein kinase II subunit alpha Participates in Wnt signaling.	+
PPARBP	Peroxisome proliferator-activated receptor-binding protein Essential for embryogenesis. Plays a role in transcriptional coactivation	++
CSK	Tyrosine-protein kinase CSK Negative regulation of cell proliferation	++
KIN27	Protein kinase A-alpha ATP binding and protein serine/threonine kinase activity	-
CUL1	Cullin-1 Mediates the ubiquitination of proteins involved in cell cycle progression, signal transduction and transcription	++
DKFZP686I18166	Hypothetical protein ATP binding and protein kinase activity	-
STAT5B	Signal transducer and activator of transcription 5B Signal transduction and activation of transcription	++
MCM7	DNA replication licensing factor MCM7 Required for DNA replication and cell proliferation. Required for S-phase checkpoint activation upon UV-induced damage.	++
SURB7	Mediator of RNA polymerase II transcription subunit 21 Regulation of transcription.	-
MST1R	Macrophage-stimulating protein receptor [Precursor] Receptor for macrophage stimulating protein (MSP). Tyrosine-protein kinase activity.	++
KHDRBS1	KH domain-containing, RNA-binding, signal transduction-associated protein 1 Role in G2-M progression in the cell cycle.	++
SYK	Tyrosine-protein kinase SYK Positive effector of BCR-stimulated responses.	++
KDR	Kinase insert domain receptor Kinase activity and receptor activity.	++
NME2	Nucleoside diphosphate kinase B Major role in the synthesis of nucleoside triphosphates other than ATP.	++
POLR2B	DNA-directed RNA polymerase II 140 kDa polypeptide DNA-dependent RNA polymerase catalyzes the transcription of DNA into RNA.	+
SRF	Serum response factor SRF is a transcription factor that binds to the serum response element (SRE)	+

## Discussion

We analyzed the use of three different criteria for predicting cancer gene candidates and concluded that: (i) the number of interaction partners of a protein that have been previously annotated as cancer gene (i.e. the cancer linker degree) is a good indicator of the likelihood of the protein to be involved in cancer; (ii) using differences in gene expression between normal tissue and cancer identifies many known cancer genes, but many non cancer genes as well; and (iii) probabilities based on structural, functional and evolutionary properties of known cancer genes (i.e. SF-Probabilities) are useful for filtering false positives from other cancer gene prediction methods. Moreover, we implemented and evaluated a method that integrates these criteria to produce reliable lists of cancer gene candidates, obtaining a positive predictive value of 73% when using very restrictive thresholds. Finally, we provided lists of cancer gene candidates and analyzed them using literature sources and information from public repositories, showing that our predictions are reliable.

Most methods used for predicting or prioritizing cancer gene candidates are biased towards genes that are well annotated and/or familiar to the researcher. This leaves unexplored many potential cancer gene candidates. However, high throughput genomic and proteomic work has now yielded relatively unbiased, although noisy, genome- and proteome-wide data sets. For example, expression studies produce large lists of over- and under-expressed genes, which are then prioritized by their differential expression rank, usually with help of a limited number of literature searches. Our integrative approach to finding cancer gene candidates can be used to obtain unbiased lists of cancer gene candidates by using the cancer linker degree of proteins to filter expression studies. We observed that the low positive predictive value obtained when using differential expression data alone (around 15% for most cancer types in our study) shows a four-fold increase when combined with protein-protein interaction data. We expect that further experimental study of our proposed cancer gene candidates will find useful the methodology presented in this work.

Separately, each of the criterion presented here for cancer gene candidate prediction has its limitations. First, methods based on protein interaction networks are limited by the fact that many cancers are the result of perturbations in the regulation of genes, which is not captured by protein-protein interaction data. Second, differential expression based methods have the drawback that many differentially expressed genes are not a cause for the cancer but rather a consequence of it. Besides, we are mapping expression levels of mRNA onto a network of protein interactions. However, it is known that the mRNA expression levels do not always match the protein expression levels [52]. Finally, methods based on structural, functional and evolutionary properties are very dependent on existing functional annotations (e.g. available GO information for a given protein) and their predictions are more stochastic than based on biological observations. These limitations could be avoided by the use of types of information such as gene regulatory networks [53] and gene copy-number alterations [7]. Moreover, recently developed experimental techniques promise an increase in the amount and types of data available [33], including protein post-translational modifications [54], tissue localization [55] and protein expression in specific cancers [56]. Finally, the integrative approach is constrained by the limitations of each independent method. However, depending on the context of application, these limitations can be avoided by ignoring irrelevant data: for example, SF-Probabilities should not be used when searching for cancer genes of unknown function.

Our reported performance results on the use of SF-Probabilities differ markedly from the evaluation presented by Lopez-Bigas and coworkers [37]. We attribute this difference to two factors: (i) we used a more extensive set of known cancer genes; (ii) we used different evaluation metrics and methods: for example, Lopez-Bigas and coworkers used a balanced dataset to evaluate their method, whereas we considered as non-cancer gene any gene that was not a known cancer gene. We believe that the performance metrics and evaluation method used in this work are more representative of predictions done on the full human genome.

The methods presented here were evaluated by comparing their cancer gene predictions with a curated list of oncogenes, tumor suppressors and stability genes [11]. This list of known cancer genes attempts to be as comprehensive as possible, but two possible biases arise from it: (i) not all methods cover the space of cancer genes to the same extent (e.g. the model used to calculate SF-Probabilities was trained on genes for which mutations have been causally implicated in cancer); and (ii) the method based on protein interaction networks heavily relies on the initial set of seed cancer genes and thus, genes isolated in the cancer network will never be pinpointed. An alternative approach to seeding our method with a list of known cancer genes is one where the seeds for building the protein interaction network are cancer-related proteins obtained with low-throughput experimental methods [57,58]. This would remove the bias introduced by the input list of known cancer genes.

## Conclusion

We showed that the integration of multiple sources of data is more reliable for predicting cancer genes than the use of one single criterion. For example, differential expression studies could benefit from the use of protein-protein interaction data to further validate their results: in the best case scenario, combining the cancer linker degree of a protein with differential expression data increased from 17% to 73% the fraction of known cancer genes within the cancer gene candidates. In conclusion, systems capable of integrating all available sources of data are fundamental to the discovery of proteins involved in cancer.

## Methods

### Known cancer genes

We downloaded cancer genes from the Memorial Sloan Kettering computational biology website CancerGenes [59] as of January 2007. We collected a set of known cancer genes by querying the website for "oncogene", "tumor suppressor" and "stability". This list comprised 1,256 cancer genes, in particular 385 oncogenes, 471 tumor suppressors and 494 stability genes (several genes belonged to more than one category).

### Protein Interaction Data

We used PIANA [38] to integrate human protein interaction data from DIP 2007.02.19 [60], MIPS 2007.04.03 [61], HPRD v6.01 [41], BIND 2007.04.03 [62], IntAct 2007.04.23 [63], BioGrid v2.026 [64] and MINT 2007.04.05 [65]. The integration of different sources of interactions into a single database allowed us to work with an extensive set of 110,457 human interactions between 36,900 different protein sequences. This set of human interaction data includes 24,812 interactions from yeast two-hybrid assays, 13,256 interactions from immunoprecipitation methods and 11,174 interactions from affinity chromatography methods. HPRD, a database manually curated from literature sources contained 38,762 interactions.

PIANA represents the protein interaction data as a network where the nodes are proteins and the edges interactions between the proteins. In such a network, a set of proteins linked to protein p_j _(ie, physically interacting with p_j_) is named "partners of p_j_". PIANA builds the network by retrieving direct interaction partners for an initial set of seed proteins (i.e. the proteins of interest).

### Expression data

We manually searched for gene expression studies between normal tissue and cancer in Oncomine [39], a cancer profiling database. We downloaded lists of over- and under-expressed genes from a total of 24 Oncomine studies, corresponding to 12 different cancer types (see additional file 6 for the list of experiments, the cancer type category assigned to them, and the total number of over- and under-expressed genes in each experiment). A gene was considered to have a significant differential expression if its Q value was lower than 0.05. Q values are assigned in Oncomine by correcting for multiple hypothesis testing the *p*-values calculated using Student's *t*-test for two-class differential expression analyses. A detailed description of the normalization process and statistical tests used in Oncomine can be found in [36,39].

### Probabilities of being cancer-gene based on structural and functional properties

We used the probabilities of being a cancer gene calculated in [37] for human genes. These probabilities were obtained using a Bayesian classification model that scored human genes for their likelihood of involvement in cancer according to structural, functional and evolutionary properties. Specifically, Lopez-Bigas and coworkers [37] relied on GO annotations [46] and sequence properties such as the extent of conservation, paralogy, and the lengths of proteins and genes. We refer to these estimated probabilities as SF-Probabilities. 12,194 human genes had an associated SF-Probability, 240 of which had been used to train the Bayesian model. 706 human genes had an SF-Probability higher than 0.95, and the SF-Probability was lower than 0.1 for 6288 human genes. Finally, 758 genes did not have an associated protein sequence in PIANA and thus, were not used in this work.

### Genes, proteins and identifiers

We used PIANA [38] to map expression data and SF-Probabilities onto the interaction network, in particular gene symbols coming from Oncomine expression studies and Ensembl identifiers coming from [37]. Throughout the text, we use the term 'cancer gene' to refer to any gene or protein involved in cancer.

### Evaluating the use of protein interaction networks to predict cancer genes

The cancer protein interaction network was built using PIANA [38] by setting the list of known cancer genes as seeds (see "protein interaction data", Material and Methods). In this network, we define the cancer linker degree (CLD) of a protein as the number of cancer genes to which it is directly connected (Figure 1). The CLD was calculated for each protein and proteins were binned by their CLDs. In this context, and given a CLD threshold of *N*, positives are proteins with CLD ≥ *N*. True positives are known cancer genes among positives. False negatives are known cancer genes whose CLD is lower than *N*. The positive predictive value is defined as the ratio between true positives and positives. Sensitivity is the ratio between true positives and the sum of false negatives and true positives. Positive predictive values and sensitivities are shown in Figure 2 for CLD thresholds with at least 5 positives.

### Evaluating the use of differential expression data to predict cancer genes

We calculated how many over- or under-expressed genes were known cancer genes for each cancer type described on Additional file 6. Moreover, we tested how many genes differentially expressed in at least 1–5 cancer types were known cancer genes. In this context, any differentially expressed gene is considered a positive. Among positives, we define as true positives those that are known cancer genes. False negatives are known cancer genes not found differentially expressed. Besides, we evaluated the prediction of cancer genes based on the differential expression rank of the cancer gene candidates in the lists of over- and under-expressed genes from Oncomine [39]. In particular, we analyzed the enrichment of cancer genes among the 50 most differentially expressed genes in the lists of over- and under-expressed genes, and compared it to the enrichment of cancer genes among all differentially expressed genes.

### Evaluating the use of structural, functional and evolutionary properties to predict cancer genes

At any given SF-Probability threshold, positives are proteins with a SF-Probability above or equal to that threshold. Among positives, true positives are those that are known cancer genes. False negatives are known cancer genes not found above the SF-Probability threshold. Genes used for training the model in [37] were discarded for the evaluation.

### Protein functions, pathways and literature

We manually analyzed cancer gene predictions from Table 4 by examining (i) the protein function and description as defined in UniProt [44]; (ii) the pathways in which the protein participated according to Reactome [45]; (iii) the molecular function and biological process as classified in the Gene Ontology (GO) [46]; and (iv) published articles retrieved using iHop [43].

### Statistical tests

The assessment on whether two binomial samples of observations are significantly different was calculated using Fisher's exact test on a 2 × 2 contingency table comparing the number of cancer genes and non-cancer genes between two groups (e.g. CLD ≥ 10 versus CLD ≥ 1). The assessment on whether a distribution of averages on the number of cancer genes calculated on random samples is significantly different from a given ratio of cancer genes was calculated using the Wilcoxon signed rank test (e.g. ratio of cancer genes found on the 5537 proteins with CLD ≥ 1 versus 1000 averages extracted from random samples of size 5537). The assessment on whether two non-Gaussian samples of observations (SF-Probabilities or number of cancer types grouped by proteins with the same CLD) come from the same distribution was calculated using the Mann-Whitney U two-sided test. Differences in the observations were considered significant for p-values lower than 0.05. All tests were performed using the implementation provided by R [66].

## Availability and Requirements

We provide the complete list of human genes with the corresponding cancer gene prediction scores according to each type of data at .

## Authors' contributions

RA conceived of the idea and performed research; BO and CS provided scientific guidance. RA drafted the manuscript. BO helped to draft the manuscript. All authors read and approved the final manuscript.

## Supplementary Material

Additional file 1 Positive predictive value and Sensitivity obtained when predicting cancer genes based on cancer linker degree of proteins measured on the cancer protein interaction network built from all interactions in PIANA, where the cancer protein interaction network has been built from the cancer gene list obtained from randomly removing 10%, 25%, 50% and 75% of genes from the complete list of known cancer genes. Click here for file

Additional file 2 Positive predictive value and Sensitivity obtained when predicting cancer genes based on cancer linker degree of proteins measured on the cancer protein interaction network built from all interactions in PIANA, where the cancer protein interaction network has been built from the cancer gene list obtained from Aouacheria et al. [40]. Click here for file

Additional file 3 **Positive predictive value and Sensitivity obtained when predicting cancer genes based on cancer linker degree of proteins measured on the cancer protein interaction network built from high-throughput interactions in PIANA**. High-throughput interactions were obtained by querying PIANA to retrieve all interactions detected by means of yeast two hybrid and affinitity purification systems. Click here for file

Additional file 4 **Positive predictive value and Sensitivity obtained when predicting cancer genes based on cancer linker degree of proteins measured on the cancer protein interaction network built from all interactions in PIANA except for those coming from the Human Protein Reference Database (HPRD)**. HPRD is a manually curated database with interactions extracted from literature [41]. By excluding from the analysis the 38,372 interactions retrieved from HPRD we were able to test the potential bias introduced by the use of interactions reported in the literature. We observed no literature bias, as both the positive predictive value and sensitivity do not significantly vary with respect to those obtained when using all interactions in PIANA (Figure 2). The positive predictive value and sensitivity shown are for accumulative cancer linker degrees (CLD) (i.e. cancer linker degree 5 represents proteins with CLD ≥ 5). The average protein in the data set is represented by CLD 0. Click here for file

Additional file 5 **Positive predictive value and Sensitivity obtained when predicting cancer genes based on the total number of interaction partners of a protein**. We observed a clear increase of involvement in cancer for proteins with many interaction partners with respect to those with just a few partners. However, the total number of partners of a protein is a worse indicator of being a cancer gene than the cancer linker degree of a protein (Figure 2). The positive predictive value and sensitivity shown are for accumulative numbers of partners (i.e. 'number of partners' 5 represents all proteins with 5 or more partners). Positive predictive value and sensitivity are shown for numbers of interaction partners with at least 5 positives. Click here for file

Additional file 6 **Gene expression studies considered for this work**. All 24 studies were downloaded from Oncomine [39]. The studies were manually grouped in 12 different cancer types. The number of over- and under-expressed genes is shown for each cancer type. Click here for file

Additional file 7 **Table with all cancer gene candidates**. For each human gene where at least one data type indicated relationship to cancer, this table shows the cancer linker degree (CLD), the number of cancer types in which it appears differentially expressed and its probability of being a cancer gene according to structural, functional and evolutionary properties (SF-Probability). Click here for file

Additional file 8 **Sources of information for analysis of candidate cancer genes in Table **4**of the article**. For each cancer gene candidate in Table 4 of the article, we reference one or more recent articles where the candidate has been linked to cancer. Information for all proteins was as well retrieved from UniProt [44], Reactome [45], GO [46] and from the literature using iHop [43]. Click here for file
