# Supplementary figures and images for: Predicting cancer involvement of genes from heterogeneous data
Source: BMC Bioinformatics. 2008 Mar 27;9:172. doi: 10.1186/1471-2105-9-172 (PMC2330045; doi:10.1186/1471-2105-9-172)

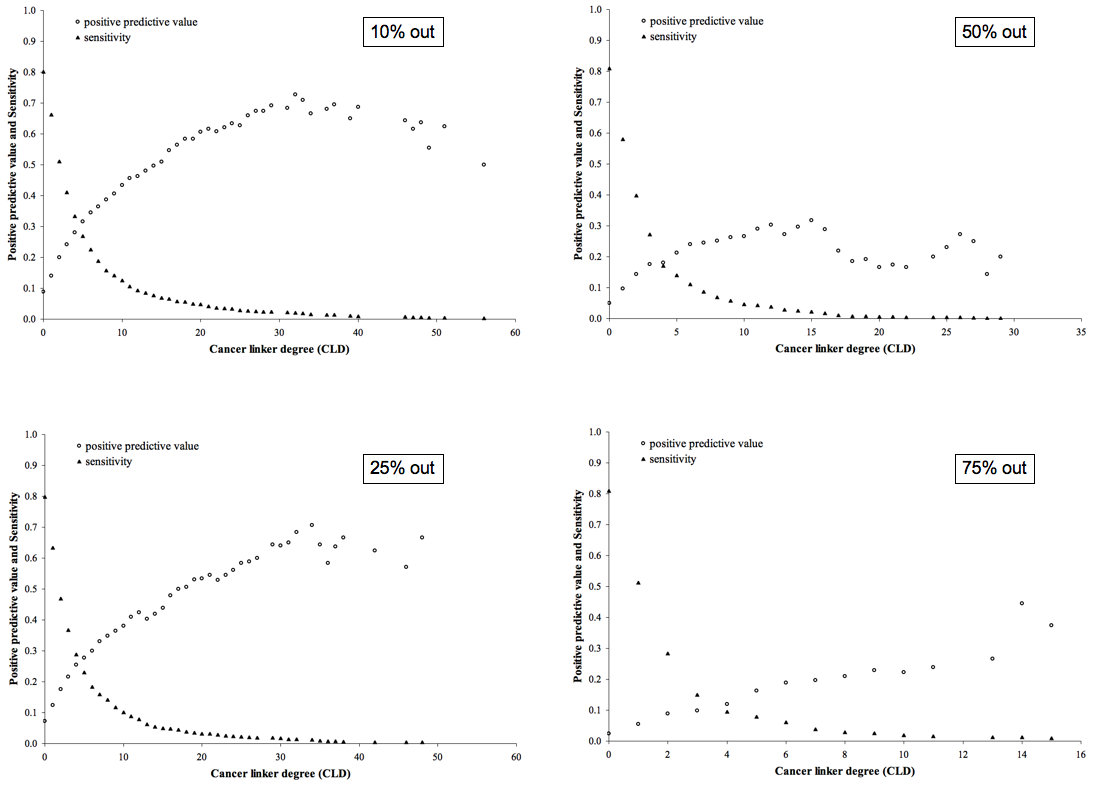

Supplement: Additional file 1 — Positive predictive value and Sensitivity obtained when predicting cancer genes based on cancer linker degree of proteins measured on the cancer protein interaction network built from all interactions in PIANA, where the cancer protein interaction network has been built from the cancer gene list obtained from randomly removing 10%, 25%, 50% and 75% of genes from the complete list of known cancer genes. [file 1471-2105-9-172-S1.tiff]

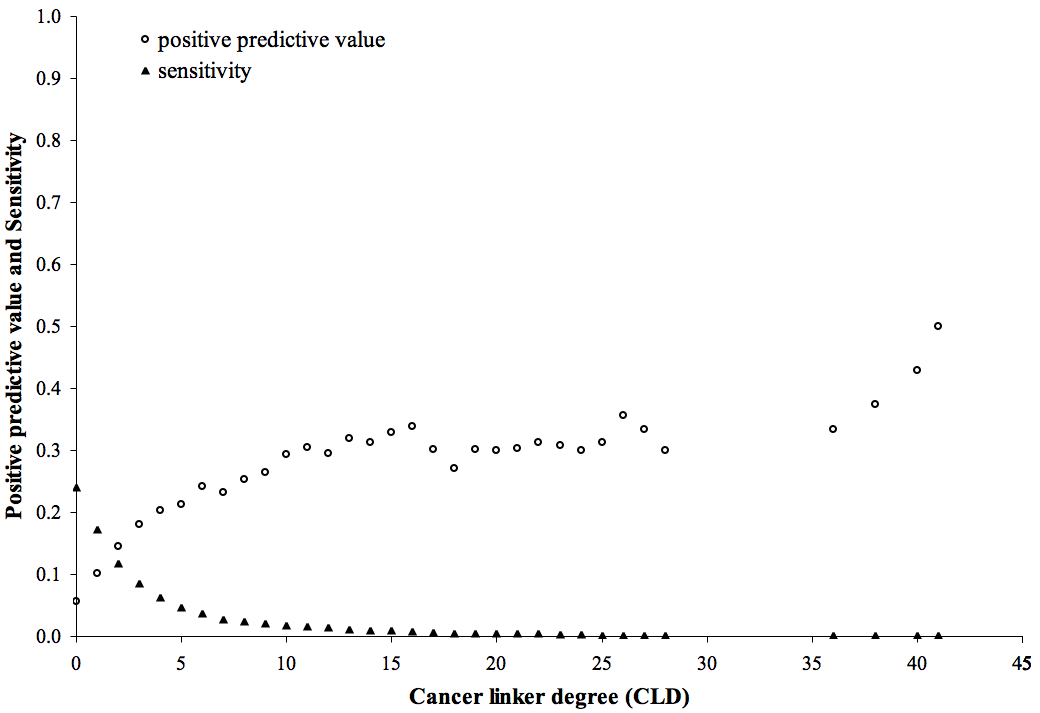

Supplement: Additional file 2 — Positive predictive value and Sensitivity obtained when predicting cancer genes based on cancer linker degree of proteins measured on the cancer protein interaction network built from all interactions in PIANA, where the cancer protein interaction network has been built from the cancer gene list obtained from Aouacheria et al. [40]. [file 1471-2105-9-172-S2.tiff]

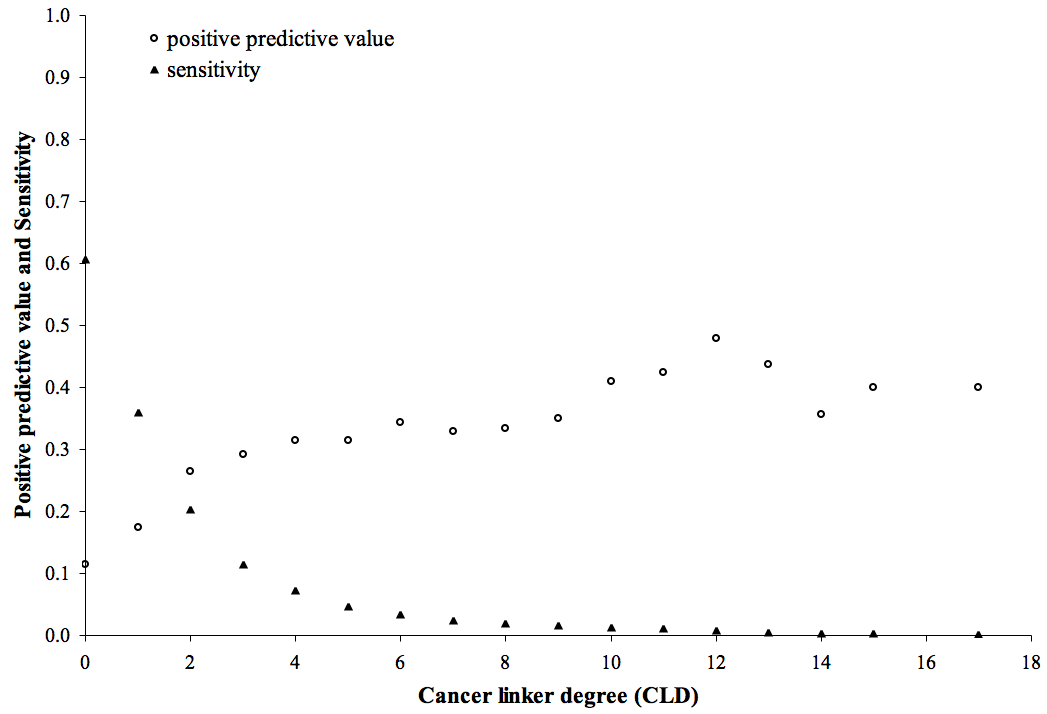

Supplement: Additional file 3 — Positive predictive value and Sensitivity obtained when predicting cancer genes based on cancer linker degree of proteins measured on the cancer protein interaction network built from high-throughput interactions in PIANA. High-throughput interactions were obtained by querying PIANA to retrieve all interactions detected by means of yeast two hybrid and affinitity purification systems. [file 1471-2105-9-172-S3.tiff]

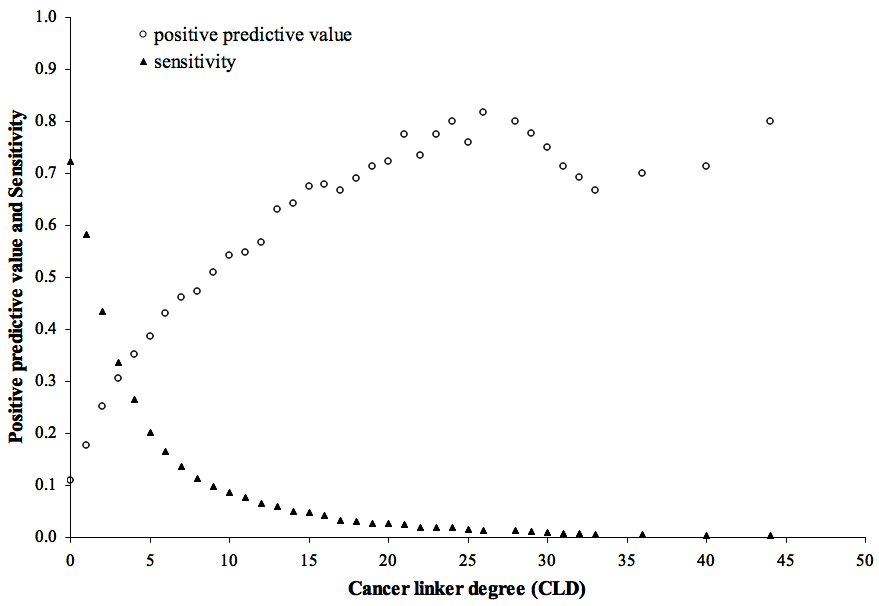

Supplement: Additional file 4 — Positive predictive value and Sensitivity obtained when predicting cancer genes based on cancer linker degree of proteins measured on the cancer protein interaction network built from all interactions in PIANA except for those coming from the Human Protein Reference Database (HPRD). HPRD is a manually curated database with interactions extracted from literature [41]. By excluding from the analysis the 38,372 interactions retrieved from HPRD we were able to test the potential bias introduced by the use of interactions reported in the literature. We observed no literature bias, as both the positive predictive value and sensitivity do not significantly vary with respect to those obtained when using all interactions in PIANA (Figure 2). The positive predictive value and sensitivity shown are for accumulative cancer linker degrees (CLD) (i.e. cancer linker degree 5 represents proteins with CLD ≥ 5). The average protein in the data set is represented by CLD 0. [file 1471-2105-9-172-S4.tiff]

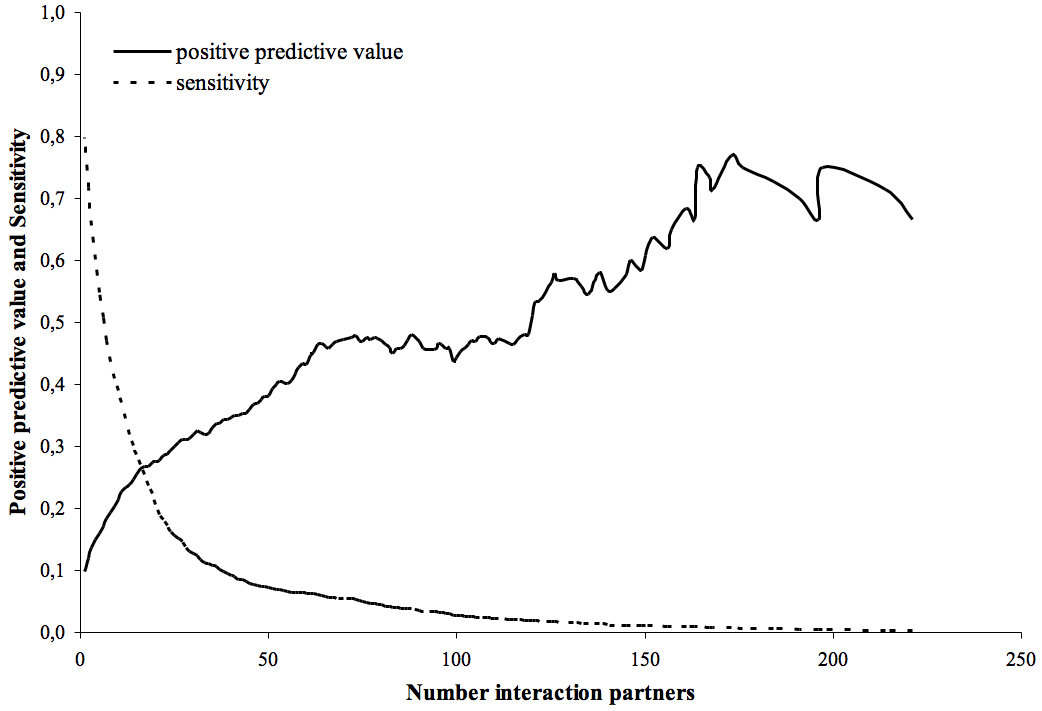

Supplement: Additional file 5 — Positive predictive value and Sensitivity obtained when predicting cancer genes based on the total number of interaction partners of a protein. We observed a clear increase of involvement in cancer for proteins with many interaction partners with respect to those with just a few partners. However, the total number of partners of a protein is a worse indicator of being a cancer gene than the cancer linker degree of a protein (Figure 2). The positive predictive value and sensitivity shown are for accumulative numbers of partners (i.e. 'number of partners' 5 represents all proteins with 5 or more partners). Positive predictive value and sensitivity are shown for numbers of interaction partners with at least 5 positives. [file 1471-2105-9-172-S5.tiff]
