# Supplementary material for: Predicting cancer involvement of genes from heterogeneous data
Source: BMC Bioinformatics. 2008 Mar 27;9:172. doi: 10.1186/1471-2105-9-172 (PMC2330045; doi:10.1186/1471-2105-9-172)
Supplement: Additional file 6 — Gene expression studies considered for this work. All 24 studies were downloaded from Oncomine [39]. The studies were manually grouped in 12 different cancer types. The number of over- and under-expressed genes is shown for each cancer type. [file 1471-2105-9-172-S6.pdf]

Oncomine study name	Study type	Cancer type	# over expressed genes	# under expressed genes
Liang brain [1]	Brain: normal vs glioblastoma	brain_glioblastoma	707	682
Shai brain [2]	Brain: white matter vs glioblastoma	brain_glioblastoma	2142	2518
Sorlie breast [3]	Breast: bening vs carcinoma	breast_carcinoma	364	251
Perou breast [4]	Breast: normal fibroadenoma vs carcinoma	breast_carcinoma	278	180
Alon colon [5]	Colon: normal vs adenocarcinoma	colon_adenocarcinoma	193	157
Notterman colon [6]	Colon: normal vs adenocarcinoma	colon_adenocarcinoma	488	319
Mutter endometrium [7]	Endometrium: normal vs adenocarcinoma	endometrium_adenocarcinoma	439	557
Powerl lung [8]	Lung: non malignant vs adenocarcinoma	lung_adenocarcinoma	92	205
Beer lung [9]	Lung: non neoplastic vs adenocarcinoma	lung_adenocarcinoma	1043	1274
Garber lung [10]	Lung: normal vs adenocarcinoma	lung_adenocarcinoma	1940	1477
Alizadeh lymphoma [11]	Lymphoma: bening vs diffuse B cell lymphoma	lymphoma_diffuseB	1125	979
Rosenwald lymphoma [12]	Lymphoma: normal blood & normal germinal vs diffuse B cell lymphoma	lymphoma_diffuseB	1011	628
Chen liver [13]	Non tumor vs carcinoma	liver_carcinoma	4192	3839
Lancaster ovarian [14]	Ovarian: normal vs adenocarcinoma	ovarian_adenocarcinoma	584	925
Welsh ovarian [15]	Ovarian: normal vs adenocarcinoma	ovarian_adenocarcinoma	566	951
Logsdon pancreas [16]	Pancreas: normal pancreatitis vs adenocarcinoma	pancreas_adenocarcinoma	647	739
Iacobuzio-donahue pancreas 2 [17]	Pancreas: normal vs adenocarcinoma	pancreas_adenocarcinoma	820	147
Latulippe prostate [18]	Prostate: non neoplastic vs carcinoma	prostate_carcinoma	288	423
Singh prostate [19]	Prostate: normal vs carcinoma	prostate_carcinoma	483	252
Dhanasekaran prostate [20]	Prostate: normal prostatic vs cancer	prostate_carcinoma	1201	959
Lapointe prostate [21]	Prostate: normal vs cancer	prostate_carcinoma	3628	3582
Welsh prostate [22]	Prostate: normal vs cancer	prostate_carcinoma	870	1311
Lenburg renal [23]	Renal: normal vs clear cell carcinoma	renal_clearCellCarcinoma	1839	2299
FriersonHF salivary gland [24]	Salivary gland: normal vs carcinoma	salivaryGland_carcinoma	1372	1370

1. Liang Y, Diehn M, Watson N, Bollen AW, Aldape KD, Nicholas MK, Lamborn KR, Berger MS, Botstein D, Brown PO *et al*: **Gene expression profiling reveals molecularly and clinically distinct subtypes of glioblastoma multiforme**. *Proc Natl Acad Sci U S A* 2005, **102**(16):5814-5819.

2. Shai R, Shi T, Kremen TJ, Horvath S, Liau LM, Cloughesy TF, Mischel PS, Nelson SF: **Gene expression profiling identifies molecular subtypes of gliomas**. *Oncogene* 2003, **22**(31):4918-4923.
3. Sorlie T, Perou CM, Tibshirani R, Aas T, Geisler S, Johnsen H, Hastie T, Eisen MB, van de Rijn M, Jeffrey SS *et al*: **Gene expression patterns of breast carcinomas distinguish tumor subclasses with clinical implications**. *Proc Natl Acad Sci U S A* 2001, **98**(19):10869-10874.
4. Perou CM, Jeffrey SS, van de Rijn M, Rees CA, Eisen MB, Ross DT, Pergamenschikov A, Williams CF, Zhu SX, Lee JC *et al*: **Distinctive gene expression patterns in human mammary epithelial cells and breast cancers**. *Proc Natl Acad Sci U S A* 1999, **96**(16):9212-9217.
5. Alon U, Barkai N, Notterman DA, Gish K, Ybarra S, Mack D, Levine AJ: **Broad patterns of gene expression revealed by clustering analysis of tumor and normal colon tissues probed by oligonucleotide arrays**. *Proc Natl Acad Sci U S A* 1999, **96**(12):6745-6750.
6. Notterman DA, Alon U, Sierk AJ, Levine AJ: **Transcriptional gene expression profiles of colorectal adenoma, adenocarcinoma, and normal tissue examined by oligonucleotide arrays**. *Cancer Res* 2001, **61**(7):3124-3130.
7. Mutter GL, Baak JP, Fitzgerald JT, Gray R, Neuberg D, Kust GA, Gentleman R, Gullans SR, Wei LJ, Wilcox M: **Global expression changes of constitutive and hormonally regulated genes during endometrial neoplastic transformation**. *Gynecol Oncol* 2001, **83**(2):177-185.
8. Powell CA, Spira A, Derti A, DeLisi C, Liu G, Borczuk A, Busch S, Sahasrabudhe S, Chen Y, Sugarbaker D *et al*: **Gene expression in lung adenocarcinomas of smokers and nonsmokers**. *Am J Respir Cell Mol Biol* 2003, **29**(2):157-162.
9. Beer DG, Kardia SL, Huang CC, Giordano TJ, Levin AM, Misek DE, Lin L, Chen G, Gharib TG, Thomas DG *et al*: **Gene-expression profiles predict survival of patients with lung adenocarcinoma**. *Nat Med* 2002, **8**(8):816-824.
10. Garber ME, Troyanskaya OG, Schluens K, Petersen S, Thaesler Z, Pacyna-Gengelbach M, van de Rijn M, Rosen GD, Perou CM, Whyte RI *et al*: **Diversity of gene expression in adenocarcinoma of the lung**. *Proc Natl Acad Sci U S A* 2001, **98**(24):13784-13789.
11. Alizadeh AA, Eisen MB, Davis RE, Ma C, Lossos IS, Rosenwald A, Boldrick JC, Sabet H, Tran T, Yu X *et al*: **Distinct types of diffuse large B-cell lymphoma identified by gene expression profiling**. *Nature* 2000, **403**(6769):503-511.
12. Rosenwald A, Wright G, Chan WC, Connors JM, Campo E, Fisher RI, Gascoyne RD, Muller-Hermelink HK, Smeland EB, Giltnane JM *et al*: **The use of molecular profiling to predict survival after chemotherapy for diffuse large-B-cell lymphoma**. *N Engl J Med* 2002, **346**(25):1937-1947.
13. Chen X, Cheung ST, So S, Fan ST, Barry C, Higgins J, Lai KM, Ji J, Dudoit S, Ng IO *et al*: **Gene expression patterns in human liver cancers**. *Mol Biol Cell* 2002, **13**(6):1929-1939.
14. Lancaster JM, Dressman HK, Whitaker RS, Havrilesky L, Gray J, Marks JR, Nevins JR, Berchuck A: **Gene expression patterns that characterize advanced stage serous ovarian cancers**. *J Soc Gynecol Investig* 2004, **11**(1):51-59.

15. Welsh JB, Zarrinkar PP, Sapinoso LM, Kern SG, Behling CA, Monk BJ, Lockhart DJ, Burger RA, Hampton GM: **Analysis of gene expression profiles in normal and neoplastic ovarian tissue samples identifies candidate molecular markers of epithelial ovarian cancer**. *Proc Natl Acad Sci U S A* 2001, **98**(3):1176-1181.
16. Logsdon CD, Simeone DM, Binkley C, Arumugam T, Greenson JK, Giordano TJ, Misek DE, Kuick R, Hanash S: **Molecular profiling of pancreatic adenocarcinoma and chronic pancreatitis identifies multiple genes differentially regulated in pancreatic cancer**. *Cancer Res* 2003, **63**(10):2649-2657.
17. Iacobuzio-Donahue CA, Maitra A, Olsen M, Lowe AW, van Heek NT, Rosty C, Walter K, Sato N, Parker A, Ashfaq R *et al*: **Exploration of global gene expression patterns in pancreatic adenocarcinoma using cDNA microarrays**. *Am J Pathol* 2003, **162**(4):1151-1162.
18. LaTulippe E, Satagopan J, Smith A, Scher H, Scardino P, Reuter V, Gerald WL: **Comprehensive gene expression analysis of prostate cancer reveals distinct transcriptional programs associated with metastatic disease**. *Cancer Res* 2002, **62**(15):4499-4506.
19. Singh D, Febbo PG, Ross K, Jackson DG, Manola J, Ladd C, Tamayo P, Renshaw AA, D'Amico AV, Richie JP *et al*: **Gene expression correlates of clinical prostate cancer behavior**. *Cancer Cell* 2002, **1**(2):203-209.
20. Dhanasekaran SM, Barrette TR, Ghosh D, Shah R, Varambally S, Kurachi K, Pienta KJ, Rubin MA, Chinnaiyan AM: **Delineation of prognostic biomarkers in prostate cancer**. *Nature* 2001, **412**(6849):822-826.
21. Lapointe J, Li C, Higgins JP, van de Rijn M, Bair E, Montgomery K, Ferrari M, Egevad L, Rayford W, Bergerheim U *et al*: **Gene expression profiling identifies clinically relevant subtypes of prostate cancer**. *Proc Natl Acad Sci U S A* 2004, **101**(3):811-816.
22. Welsh JB, Sapinoso LM, Su AI, Kern SG, Wang-Rodriguez J, Moskaluk CA, Frierson HF, Jr., Hampton GM: **Analysis of gene expression identifies candidate markers and pharmacological targets in prostate cancer**. *Cancer Res* 2001, **61**(16):5974-5978.
23. Lenburg ME, Liou LS, Gerry NP, Frampton GM, Cohen HT, Christman MF: **Previously unidentified changes in renal cell carcinoma gene expression identified by parametric analysis of microarray data**. *BMC Cancer* 2003, **3**:31.
24. Frierson HF, Jr., El-Naggar AK, Welsh JB, Sapinoso LM, Su AI, Cheng J, Saku T, Moskaluk CA, Hampton GM: **Large scale molecular analysis identifies genes with altered expression in salivary adenoid cystic carcinoma**. *Am J Pathol* 2002, **161**(4):1315-1323.
