# Supplementary material for: Predicting cancer involvement of genes from heterogeneous data
Source: BMC Bioinformatics. 2008 Mar 27;9:172. doi: 10.1186/1471-2105-9-172 (PMC2330045; doi:10.1186/1471-2105-9-172)
Supplement: Additional file 8 — Sources of information for analysis of candidate cancer genes in Table 4of the article. For each cancer gene candidate in Table 4 of the article, we reference one or more recent articles where the candidate has been linked to cancer. Information for all proteins was as well retrieved from UniProt [44], Reactome [45], GO [46] and from the literature using iHop [43]. [file 1471-2105-9-172-S8.pdf]

Information for all proteins was as well retrieved from UniProt [1], Reactome [41] and from the literature using iHop [2].

Gene name	References
CDK9	[3]
GATA2	[4]
ATF2	[5]
CCNB1	[6]
CSNK2A2	[7]
PPARBP	[8]
CSK	[9]
KIN27	-
CUL1	[10]
DKFZP686I18 166	-
STAT5B	[11]
MCM7	[12]
SURB7	-
MST1R	[13]
KHDRBS1 (*a.k.a.* Sam68)	[14]
SYK	[15]
KDR	[16]
NME2	[17]
POLR2B	[18]
SRF	[19]

1. **The Universal Protein Resource (UniProt)**. *Nucleic Acids Res* 2007, **35**(Database issue):D193-197.
2. Hoffmann R, Valencia A: **A gene network for navigating the literature**. *Nature genetics* 2004, **36**(7):664.
3. Shan B, Zhuo Y, Chin D, Morris CA, Morris GF, Lasky JA: **Cyclin-dependent kinase 9 is required for tumor necrosis factor-alpha-stimulated matrix metalloproteinase-9 expression in human lung adenocarcinoma cells**. *The Journal of biological chemistry* 2005, **280**(2):1103-1111.
4. Li Z, Godinho FJ, Klusmann JH, Garriga-Canut M, Yu C, Orkin SH: **Developmental stage-selective effect of somatically mutated leukemogenic transcription factor GATA1**. *Nature genetics* 2005, **37**(6):613-619.
5. Liu H, Deng X, Shyu YJ, Li JJ, Taparowsky EJ, Hu CD: **Mutual regulation of c-Jun and ATF2 by transcriptional activation and subcellular localization**. *The EMBO journal* 2006, **25**(5):1058-1069.
6. Zhao M, Kim YT, Yoon BS, Kim SW, Kang MH, Kim SH, Kim JH, Kim JW, Park YW: **Expression profiling of cyclin B1 and D1 in cervical carcinoma**. *Experimental oncology* 2006, **28**(1):44-48.
7. Mazieres J, He B, You L, Xu Z, Jablons DM: **Wnt signaling in lung cancer**. *Cancer letters* 2005, **222**(1):1-10.

8. Zhu Y, Qi C, Jain S, Le Beau MM, Espinosa R, 3rd, Atkins GB, Lazar MA, Yeldandi AV, Rao MS, Reddy JK: **Amplification and overexpression of peroxisome proliferator-activated receptor binding protein (PBP/PPARBP) gene in breast cancer**. *Proceedings of the National Academy of Sciences of the United States of America* 1999, **96**(19):10848-10853.
9. Humar B, Fukuzawa R, Blair V, Dunbier A, More H, Charlton A, Yang HK, Kim WH, Reeve AE, Martin I *et al*: **Destabilized adhesion in the gastric proliferative zone and c-Src kinase activation mark the development of early diffuse gastric cancer**. *Cancer research* 2007, **67**(6):2480-2489.
10. Nakayama KI, Nakayama K: **Ubiquitin ligases: cell-cycle control and cancer**. *Nature reviews* 2006, **6**(5):369-381.
11. Kazansky AV, Spencer DM, Greenberg NM: **Activation of signal transducer and activator of transcription 5 is required for progression of autochthonous prostate cancer: evidence from the transgenic adenocarcinoma of the mouse prostate system**. *Cancer research* 2003, **63**(24):8757-8762.
12. Kebebew E, Peng M, Reiff E, Duh QY, Clark OH, McMillan A: **Diagnostic and prognostic value of cell-cycle regulatory genes in malignant thyroid neoplasms**. *World journal of surgery* 2006, **30**(5):767-774.
13. Welm AL, Sneddon JB, Taylor C, Nuyten DS, van de Vijver MJ, Hasegawa BH, Bishop JM: **The macrophage-stimulating protein pathway promotes metastasis in a mouse model for breast cancer and predicts poor prognosis in humans**. *Proceedings of the National Academy of Sciences of the United States of America* 2007, **104**(18):7570-7575.
14. Paronetto MP, Achsel T, Massiello A, Chalfant CE, Sette C: **The RNA-binding protein Sam68 modulates the alternative splicing of Bcl-x**. *The Journal of cell biology* 2007, **176**(7):929-939.
15. Yuan Y, Wang J, Li J, Wang L, Li M, Yang Z, Zhang C, Dai JL: **Frequent epigenetic inactivation of spleen tyrosine kinase gene in human hepatocellular carcinoma**. *Clinical cancer research* 2006, **12**(22):6687-6695.
16. Forsti A, Jin Q, Altieri A, Johansson R, Wagner K, Enquist K, Grzybowska E, Pamula J, Pekala W, Hallmans G *et al*: **Polymorphisms in the KDR and POSTN genes: association with breast cancer susceptibility and prognosis**. *Breast cancer research and treatment* 2007, **101**(1):83-93.
17. Ouatas T, Salerno M, Palmieri D, Steeg PS: **Basic and translational advances in cancer metastasis: Nm23**. *Journal of bioenergetics and biomembranes* 2003, **35**(1):73-79.
18. Michiels S, Danoy P, Dessen P, Bera A, Boulet T, Bouchardy C, Lathrop M, Sarasin A, Benhamou S: **Polymorphism discovery in 62 DNA repair genes and haplotype-associations with risks for lung, and head and neck cancers**. 2007.
19. Lee HJ, Yun CH, Lim SH, Kim BC, Baik KG, Kim JM, Kim WH, Kim SJ: **SRF is a nuclear repressor of Smad3-mediated TGF-beta signaling**. *Oncogene* 2007, **26**(2):173-185.
